# New Recommendation on Biological Materials Could Hamper Muscular Dystrophy Research

**DOI:** 10.1371/currents.md.f21a7be89ed7fcdc689bf30e97dd2756

**Published:** 2016-12-21

**Authors:** Pauline McCormack, Simon Woods

**Affiliations:** Policy, Ethics and Life Sciences Research Centre, Newcastle University, Claremont Bridge, Newcastle upon Tyne, UK; Policy, Ethics and Life Sciences Research Centre, Newcastle University, Claremont Bridge, Newcastle upon Tyne, UK

## Abstract

The new ‘Recommendation of the Committee of Ministers to member States on research on biological materials of human origin’, adopted in Europe in May 2016 is confusing and lacks specificity on the research use of biomaterials taken from persons not able to consent. It is possible to interpret the relevant clauses in a restrictive manner and doing so would hamper biobank research, by requiring researchers or biobank curators to examine individual records in detail, to check they are adhering to the Recommendation. This would be particularly problematic for muscular dystrophy and other rare disease research, the progress of which relies increasingly on the sharing of biomaterials and data internationally, as it will add complexity to the logistics of biomaterials and data sharing and introduce barriers for researchers preparing biomaterials for sharing. Such barriers are contradictory to EC policies on promoting and funding rare disease research and removing barriers to better care and treatment. Such policies work in concert with international progress in rare disease research, in particular the NIH’s Rare Diseases Clinical Research Network and Genetic and Rare Diseases Information Centre. The rare disease community has in recent years worked to create a common framework of harmonised approaches to enable the responsible, voluntary, and secure sharing of biomaterials and data. These efforts are supported by the European Commission in such moves as FP7 funding to advance rare disease research and the introduction of National Plans for rare disease; and are bolstered by similar efforts in the USA via the Clinical and Translational Science Awards Program and the NIH/NCATS Patient Registry developments. Introducing Recommendations from the Committee of Ministers, containing clauses which are incompatible to the efforts to advance rare disease research, seems counter-productive.

## Background

The pre-amble to the new Recommendation of the Committee of Ministers to member States on research on biological materials of human origin, adopted in May 2016, announces that it is designed to take into account new developments in biobanking including the cross-border flow of such materials for research^1^. However, the recommendation contains a stipulation on materials from ‘persons not able to consent’ which is confusing and which has the potential to stifle global efforts in rare disease which aim to enable and encourage resource sharing among researchers.

Recommendation 12.1 (repeated at 22.5) states that for those persons not able to consent in law biomaterials ‘should only be obtained or stored for future research having the potential to produce, in the absence of direct benefit to the person concerned, benefit to other persons in the same age category or afflicted with the same disease or disorder or having the same condition, and if the aims of the research could not reasonably be achieved using biological materials from persons able to consent’ (ibid).

The ethical intention behind the recommendation is important because it recognises the potential vulnerability of incapacitated persons participating in research^2^. However the wording is problematic because does not indicate whether it applies to children and/or to adults who lack capacity. The inclusion of the term ‘same age category’ implies that the Recommendation is referring to children but is not explicit, which in itself in problematic as it means the Recommendation may be read as referring to children or not and therefore result in varying practices. The Recommendation does not provide a reasonable rationale for restricting the potential beneficiary groups to parity of age, nor is there any justification for limiting benefit to those with the same condition, a consideration which is particularly pertinent for rare disease research. In addition it does not distinguish sufficiently between the ethical concerns which arise at the point of entering a person who lacks capacity into a study in which tissue will be collected, from the ethical issues of using tissue already collected and stored. Most important here is that some consideration is given to the potential for risk and burden from the research procedure quite independent of whether the use of tissue has the potential to benefit others. Without clarification on these points the recommendation is likely to be interpreted in a restrictive way, for example by a researcher deciding that the Recommendation does refer to children’s samples in a biobank and that those samples can not therefore be shared with research studies which include adults with a different condition. Such a move has the potential to be highly detrimental to important areas of research collaboration, particularly in rare diseases, where there is general agreement that samples should have maximum utility.

**Children or adults?**

The wording is unclear as to whether the Recommendation is intended to apply to adults and children. Although there are similarities in the ethical issues raised there are important differences; there are also differences in the legal provisions for these different contexts. This latter point is important because in most jurisdictions there are provisions which allow a role for the proxy consent of parents or a person acting with parental responsibility. The person acting with parental responsibility can bring other safeguards into the decision including their knowledge of the child and their judgement regarding the child’s best interests. When it comes to adults who lack capacity and their involvement in research then the situation is more heterogeneous with far fewer jurisdictions providing specific legal provision for research. Many jurisdictions do not recognise a role for substituted judgement or proxy consent for adults which has prompted the development of specific legislation to deal with adults who lack capacity. England and Wales is one jurisdiction where there is a specific law dealing with the involvement of adults who lack capacity in research. The Mental Capacity Act (MCA 2005) of England and Wales is a good example where there is a specific legal framework for the recruitment of adults who lack capacity and sets out the criteria, which must be met in order for a research project to proceed.

**Rare disease research**

The scarcity and therefore elevated value of biological materials for research in rare diseases provides an argument for making use of all available materials and biobank legislation usually provides a robust legal framework for this. Of particular importance for rare disease research is the capacity for the international sharing of biological materials. This is an important consideration when recognising the right of people living with a rare disease to benefit from health care, prevention, and medical treatment.

This imperative is recognised internationally, as can be seen by the increasing profile of, and funding around, rare disease, which includes significant input from the European Commission and the NIH. In addition, worldwide collaborations are pushing forward with initiatives which are building infrastructures and standards to ease the sharing of data and biomaterials for rare disease. The International Rare Disease Research Consortium (IRDiRC), aims to accelerate rare disease diagnosis and therapies, and the Global Alliance for Genomics and Health (GA4GH) is working to create a common framework of harmonised approaches to enable the responsible, voluntary, and secure sharing of genomic and clinical data.

All these initiatives represent a significant expenditure of resources by public and private organisations around the world and epitomise an era of collaboration for rare disease which has never been seen before and which is already bearing fruit^3^. If organisations interpret the new Recommendation as being applicable to children, this could cause extensive problems for researchers using materials from rare disease biobanks.

We have indicated the ambiguity of recommendation 12.1 but in addition there are three significant reasons why they should not be applied to children’s tissues. First, because they conflict with the UN Convention on the Rights of the Child, the thrust of which emphasises the right of children to benefit from the ‘highest attainable standard of health and to facilities for the treatment of illness and rehabilitation of health’ (1997, p7), as outlined in Article 23.

Second because it conflicts with the express, wishes of rare disease patients, families and organisations to engage in collaborative, solidaristic actions to improve rare disease research through collective endeavour across different disorders and national boundaries. A child whose future is foreclosed by a progressive and incurable disease inspires a moral claim on human endeavour, to find a means of treating and ideally curing them, that few would dispute. The vulnerability of such children might be used to justify a highly precautionary attitude to research governance but Recommendation 12.1 is restrictive for no net gain in ethical safeguards. The application of recommendation 12.1 to children would repudiate the wishes of rare disease patients and their families and be contra to the decades of creative and collective self-organisation and education that rare disease patient organisations have initiated^4^^,^ 5. From the 1960s onwards, parents, on behalf of their children, developed organisations that are: major funders of research; effective political lobbyists; and founders of strategic alliances, and part of this has been a practical and intellectual challenge to the presumption of vulnerability^6^. The authors are connected to RD Connect, an EC FP7 funded project which is constructing an integrated global platform connecting databases, registries, biobanks and clinical bioinformatics for rare disease research. Our research shows that rare disease patients and patient organisations are highly solidaristic. They recognise that unity through common interests and objectives can be a powerful lever for action and also that they may benefit from research unrelated directly to their condition, in the future^7^^,^ 8. It can not be known where this potential crossover of benefit might arise and it is therefore important not to unduly restrict research to particular disease boundaries.

Third because the recommendation represents a narrowing of what is currently permitted in other ethical guidelines/national law and is therefore contradictory to current practices. The importance of emphasising potential benefit and negligible risk, which the recommendations do not mention, is that it provides considerable safeguard to the person who lacks capacity; especially significant in the context of taking tissue samples. Whereas the emphasis given in the recommendations to the potential beneficiary being of the ‘same age category’ is too vague and of questionable ethical importance. What is an age category? Is it a five or ten or twenty year range? Is it there to prevent the exploitation of children in favour of adults? On one interpretation the Recommendation seems unduly restrictive, as tissue taken from a healthy child who does not have the capacity to consent, may be an important control for research conducted on a specific children’s disease. The Recommendations do not seem to recognise that tissue obtained for a specific research purpose is often not exhausted for that purpose and often retained for future unspecified research, equally tissue may be obtained for an unspecified broad purpose, as is commonly done in the context of biobanks. The Recommendation seem particularly restrictive on this point and is therefore of serious concern to those conducting a broad programme of research, such as those undertaking research into genetic, childhood diseases.

## Conclusions

If Recommendation 12.1 is applied to children, it would unduly restrict rare disease research by preventing the use of children’s biomaterials in research involving adults, or on anyone without the same condition and thereby constrict children’s right to benefit from the solidaristic actions of others. It would make efforts to improve data and biomaterials sharing for rare diseases more complex, time consuming and less efficient. Platforms such as RD Connect and BBMRI-ERIC which were designed to contribute to the efficacy and excellent of European research by easing access to resources, could be rendered less efficient by the need to introduce logistical complexity to isolate samples from children and to examine whether requests for samples are made in accordance with the proposed Recommendation. This is a crucial factor as 75% of rare diseases affect children and 30% of rare disease patients die before they are 5 years old.

Researchers and research organisations should be wary of a restrictive interpretation of Recommendation, lest this by default becomes soft policy. A clarification from the Council of Ministers as to whether the relevant paragraphs of the new Recommendation are meant to include biomaterials from children would be helpful and would recognise the potential ramifications of their decision for research on childhood conditions, and for the unprecedented progress in rare disease.

## Data Availability Statement

All relevant data are within the article.

## Competing Interest Statement

The authors declare that they have no competing interests.

## Corresponding Author

Pauline McCormack: pauline.mccormack@ncl.ac.uk

## Author Contributions

PMcC and SW contributed equally regarding ideas, drafting and editing of the manuscript.
